# Urodynamic Predictive Factors for Successful Treatment Outcomes Following Intravesical Botulinum Toxin a Injection in Patients with Detrusor Overactivity

**DOI:** 10.3390/biomedicines13092147

**Published:** 2025-09-03

**Authors:** Yu Khun Lee, Hann-Chorng Kuo

**Affiliations:** Department of Urology, Hualien Tzu Chi Hospital, Buddhist Tzu Chi Medical Foundation, Buddhist Tzu Chi University, Hualien 970004, Taiwan; leeyukhun@gmail.com

**Keywords:** urinary incontinence, detrusor overactivity, intravesical injection, predictive factors

## Abstract

**Purpose**: This study aimed to identify the predictive factors for successful or failed treatment outcomes following intravesical injection of botulinum toxin A (BoNT-A) through an analysis of baseline video urodynamic characteristics and parameters. **Methods**: This study retrospectively analyzed the therapeutic outcomes of intravesical BoNT-A injection in patients who had an overactive bladder (OAB), who were diagnosed with detrusor overactivity (DO), and who had been refractory to OAB medications or intolerant of the adverse events associated with them. Treatment outcomes were classified as successful, improved, or failed according to the patients’ subjective report of improvement in OAB symptoms at three months post-injection. The patients were split into male and female cohorts and neurogenic or non-neurogenic DO for data analysis. The video urodynamic study characteristics and parameters were compared across the successful, improved, and failed subgroups. **Results**: This study included 571 patients who received intravesical BoNT-A injections for treating their OAB and urodynamic DO, of which 272 were men and 299 were women. The treatment outcome of BoNT-A injection was successful in 55.7%, improved in 27.8%, and failed in 16.5% of the patients. Patients with urodynamic detrusor underactivity (DU) and neurogenic DO due to diseases of the central nervous system did not usually achieve a successful outcome. The following factors predicted successful treatment outcomes following BoNT-A injection: lower baseline detrusor pressure, higher maximum flow rate (Qmax), larger voided volume, and smaller post-void residual (PVR) in men; larger voided volume and smaller PVR in women. **Conclusions**: The therapeutic success of intravesical BoNT-A injection for treating refractory OAB can be predicted by lower Pdet, higher Qmax, larger voided volume, and smaller PVR in men and by higher Qmax and smaller PVR in women. Patients with neurogenic DO and DU usually have unsuccessful treatment outcomes.

## 1. Introduction

Urinary incontinence induced by detrusor overactivity (DO) is a commonly encountered lower urinary tract dysfunction, particularly in elderly people and patients with diseases of the central nervous system (CNS) [1]. In the overall population, the prevalence of overactive bladder (OAB) syndrome and urinary incontinence is estimated to be 12% in men and 15% in women [2]. The first-line treatment for OAB syndrome or urodynamic DO includes behavioral modification and medications such as antimuscarinic agents or beta-3 adrenoceptor agonists [3]. Although OAB medications exhibit good therapeutic efficacy, some patients do not respond well to medical treatment, and some cannot tolerate the adverse events occurring after taking OAB medications [4]. For such patients, injection of intravesical botulinum toxin A (BoNT-A) has become an emergent alternative treatment [5]. Basic and clinical research has demonstrated that a BoNT-A injection can effectively decrease detrusor contractility, attenuate urgency sensation, and increase functional bladder capacity. Moreover, the therapeutic effects of a BoNT-A injection last for 6–9 months [6].

However, BoNT-A injection for treating OAB or DO is also associated with some adverse events and failure. Although the overall success rate of BoNT-A to treat OAB or urodynamic DO was higher than 60% [7] and improved all symptoms and urodynamic parameters [8], long-term compliance with BoNT-A for treating OAB was only 25% [9]. Previous studies evaluating the treatment efficacy of BoNT-A reported that patients might develop difficulty in urination, large post-void residual (PVR) volume, acute or chronic urinary retention, and urinary tract infection after BoNT-A injection [7]. The voiding efficiency (VE) usually declined over the first month post-injection and slowly improved over the next 3 months. During this post-procedure period, patients might still experience urgency or urgency urinary incontinence (UUI) together with urinary difficulty [9]. Therefore, despite the therapeutic effect of BoNT-A on detrusor contractility, a certain percentage of patients with OAB or urodynamic DO reported that the treatment outcome failed because they experienced symptom persistence and severe dysuria. Consequently, these patients did not continue BoNT-A injection when the symptoms of OAB or DO relapse [10].

Data on the therapeutic effect and safety profile of BoNT-A in the treatment of OAB or urodynamic DO largely come from clinical trials [11,12]. In real-world practice, patients with OAB or urodynamic DO might have many underlying diseases or conditions, such as latent CNS diseases, occult bladder outlet obstruction, concomitant low detrusor contractility, or large PVR due to comorbidities at baseline [13,14]. These underlying pathologies of OAB might result in inadequate response to BoNT-A treatment, increased PVR, or the development of urinary retention [8,9,10]. Consequently, patients might be dissatisfied with the treatment outcome and reject subsequent BoNT-A injections for treating recurrent OAB symptoms [13,15]. This study aimed to identify predictive factors for successful or failed treatment outcomes by analyzing patients with DO of different subtypes and baseline videourodynamic characteristics and parameters. The results of this study could inform clinicians in the selection of appropriate patients with OAB or urodynamic DO for intravesical BoNT-A injection.

## 2. Methods

From January 2002 through December 2024, the following patients were retrospectively analyzed for the therapeutic outcome of intravesical BoNT-A injection: (i) those who had urgency with or without urgency urinary incontinence (UUI) verified by the validated questionnaire Overactive Bladder Symptom Score, (ii) those who received a diagnosis of DO, (iii) those who still bothered by the urgency or UUI after OAB medications for at least 3 months, and (iv) those who had failed to tolerate the adverse events associated with OAB medications for at least 3 months. All patients were well documented and underwent videourodynamic study (VUDS) before intravesical BoNT-A injection. This study was approved by the Institutional Review Board of the Research Ethics Committee of Hualien Tzu Chi Hospital, Buddhist Tzu Chi Medical Foundation, coded IRB 114-109-B, dated 14 June 2025. The requirement for informed consent was waived because of the nature of the retrospective analysis.

The VUDS was performed in the sitting position for women and the standing position for men. A 6-Fr double-lumen urethral catheter was inserted to infuse contrast medium containing normal saline at the rate of 20–30 mL/min and record the intravesical pressure. An 8-Fr rectal balloon catheter was inserted into the anus to record the intra-abdominal pressure. The detrusor pressure (Pdet) was obtained by subtracting the intra-abdominal pressure from the intravesical pressure during the storage and voiding phases. The maximum flow rate (Qmax) was recorded during the voiding phase, and the Pdet at Qmax was considered as the voiding Pdet. Cystometric bladder capacity, voided volume, and PVR were calculated. Concomitant urethral sphincter electromyography was also performed, and bladder outlet conditions, such as DO, detrusor underactivity (DU), detrusor acontractile (DA), bladder neck dysfunction, benign prostatic obstruction, other bladder outlet obstruction, and dysfunctional voiding, were diagnosed according to the recommendations of the International Continence Society [16]. Patients with a history of cerebrovascular accident, Parkinson’s disease, or dementia were considered to have neurogenic DO (NDO), whereas those without CNS diseases were considered to have non-NDO. Patients with NDO were included in this study only if they had a sensation of urgency and were able to void.

The procedure followed for the intravesical injection of BoNT-A has been described previously [17]. Under intravenous general anesthesia, each patient was injected with 100 U of onabotulinumtoxinA at 20 sites of the bladder wall, sparing the trigone. BoNT-A was injected into the suburothelial space to ensure that all of the solution was injected inside the bladder wall. After placing an indwelling urethral catheter overnight, the patients were discharged and followed up at the outpatient clinic at 1 week, 4 weeks, and then every month until 6 months post-injection. During the follow-up visits, the patients were subjected to uroflowmetry, PVR measurement, and urinalysis. Moreover, they were asked to report their perception regarding difficulty in urination, urgency, and UUI. Antibiotics were prescribed if a urinary tract infection was detected.

The treatment outcome for each patient was ascertained on the basis of their subjective report of the improvement of the urgency severity scale (USS) at 3 months post-injection. The treatment outcome was classified as successful if the patient became totally dry or only had mild urgency (USS = 1) without complaints of difficulties during urination. The treatment outcome was classified as improved if the patient presented improved OAB symptoms but still experienced mild-to-moderate urgency (USS = 2) or occasional UUI and mild-to-moderate difficulties during urination. The treatment outcome was classified as failed either if the patient still experienced moderate-to-severe urgency and frequent UUI (USS = 3), similar to their baseline status, regardless of the difficulty level during urination, or if they exhibited acute urinary retention (AUR), regardless of their urgency/UUI status.

The patients were split by gender into two cohorts and by non-NDO or NDO for data analysis. The VUDS characteristics and parameters were compared across patients in the successful, improved, and failed treatment outcome groups. Continuous variables are presented as mean ± standard deviation, and categorical variables are presented as counts and percentages. Group comparisons were conducted using the one-way ANOVA with Scheffé post hoc tests for continuous baseline variables and chi-square tests for categorical variables when comparing the three outcome groups. Predictive factors for treatment success were analyzed using logistic regression. The optimal value of each variable for predicting a successful treatment outcome was determined by calculating the area under the curve of a receiver operating characteristic curve. A *p* value < 0.05 was considered to denote a statistically significant difference. All analyses were conducted using SPSS software for Windows (version 16.0; Chicago, IL, USA).

## 3. Results

The study included 571 patients who received intravesical BoNT-A injection for their OAB and urodynamic DO, of which 272 were men and 299 were women. A successful treatment outcome was reported in 139 men (51.1%) and 179 women (59.9%), an improved outcome in 91 men (33.5%) and 68 women (21.7%), and a failed outcome in 42 men (15.4%) and 52 women (17.4%).

Table 1 lists the clinical demographics as well as the baseline VUDS characteristics and parameters of the male subgroups with successful, improved, and failed treatment outcomes. Men in the successful outcomes subgroup were significantly younger; had higher Qmax, voided volume, VE, and bladder contractility index (BCI); and lower PVR volume and bladder outlet obstruction index than men in the other subgroups. Men with NDO due to cerebrovascular accident, Parkinson’s disease, or dementia and those with benign prostatic obstruction, bladder neck dysfunction, and DU/DA had a lower successful outcome.

Table 2 presents the data for female patient subgroups with successful, improved, or failed treatment outcomes after BoNT-A injection. Women with NDO due to cerebrovascular accident or dementia did not have a successful treatment outcome. Women with urodynamic DU/DA; lower Pdet, Qmax, voided volume, VE, and BCI; and higher PVR volume and bladder outlet obstruction index had less favorable treatment outcomes.

Logistic regression was performed to identify predictive factors for favorable treatment outcomes in the male and female cohorts. Table 3 presents the results of univariate and multivariate analyses that were conducted to identify predictive factors for successful treatment outcomes. Successful treatment outcomes in both male and female patients were predicted by higher Qmax, voided volume, VE, and BCI and lower PVR volume and bladder outlet obstruction index. In men, lower Pdet was also a predictor for successful outcomes. The results of multivariate analysis indicated that larger voided volume and smaller PVR volume predicted successful treatment outcomes in both men and women following BoNT-A injection. In men, lower Pdet or higher Qmax also served as predictors of successful outcomes. Similar results were obtained when we examined predictive factors for unsatisfactory (improved or failed) treatment outcomes.

We calculated the area under the curve of a receiver operating characteristic curve to identify predictive factors for unsatisfactory treatment outcomes as well as their cut-off values. The results revealed that women may experience unsuccessful treatment outcomes if their baseline Qmax < 12.5 mL/s, voided volume < 229.5 mL, PVR volume > 35 mL, and VE < 0.895. The corresponding cut-off values in men were baseline Qmax < 8.5 mL/s, voided volume < 149 mL, PVR volume > 35 mL, and VE < 0.801 (Table 4).

## 4. Discussion

In this study, the treatment outcome of intravesical BoNT-A injection was successful in 55.7%, improved in 27.8%, and failed in 16.5% of patients with urodynamic DO. Although the therapeutic effect of BoNT-A was recorded in 83.5% of patients (successful and improved subgroups), a high percentage of patients did not consider the treatment to be successful because of remaining urgency/UUI, difficulties during urination, and large PVR volumes. In real-world practice, the treatment outcome of BoNT-A injection might be successful only in patients with high Qmax, large voided volumes, and small PVR volumes. Patients with urodynamic DU/DA usually cannot achieve a successful treatment outcome following BoNT-A injection.

The treatment strategy for OAB or urodynamic DO is well documented in the guidelines [3,18]. Behavioral therapy has always been the first-line treatment. Antimuscarinic agents and/or beta-3 adrenoceptor agonists have been recommended as a second-line treatment for any subtype of OAB. The therapeutic efficacy of oral OAB medication is nearly 70% [3,4]. For patients who are refractory or intolerant to oral pharmacological treatment, the use of an intravesical injection of 100 U of BoNT-A (onabotulinumtoxinA) has been well-investigated, documented, and recommended [3,12,18]. In addition to oral medication and BoNT-A injection, transcutaneous or percutaneous tibial nerve stimulation has been attempted [19]. Among the three treatment modalities, BoNT-A injection is the most effective way to treat DO [20]. Statistically significant and clinically relevant improvements in symptoms and patient-reported outcomes and tolerability were seen in patients with overactive bladder and urinary incontinence [11]. Subjectively, 86% of the patients would choose this procedure for their bladder condition again. In this study, the total rates of successful and improved treatment outcomes are similar to this result [21].

Intravesical BoNT-A injection effectively reduces the frequency of urgency/UUI episodes by inhibiting the release of acetylcholine from nerve terminals, thereby blocking motor functions [22]. In addition, evidence also indicate that BoNT-A has effect on the purinergic system and afferent sensitization resulting in sensory impairment [23]. Previous phase 3 clinical trial have demonstrated that Patients perceived a significant improvement in their condition, as measured by patients with a positive treatment response on the treatment benefit scale 62.8% for onabotulinumtoxinA versus 26.8% for placebo (*p* < 0.001) [24], and 60.8% for onabotulinumtoxinA versus 29.2% for placebo (*p* < 0.001) in another trial [25].

Because the therapeutic mechanism of BoNT-A involves both sensory and motor pathways, detrusor contractility will also be greatly impaired in the early months following BoNT-A injection. The VE will also decrease in the first 3 months following BoNT-A injection [26]. During this period, patients might experience no improvement in functional bladder capacity and the frequency of urgency/UUI episodes. Although BoNT-A has achieved its therapeutic effect by increasing bladder capacity and compliance, patients still cannot empty their bladder completely, resulting in a large PVR volume; therefore, they will regard the treatment to have failed [6,9]. These conflicting treatment effects are typically observed in patients with OAB in combination with urodynamic DU/DA, CNS diseases, or age-associated frailty [27]. In this study, successful treatment outcomes were reported only in 55.7% of patients and improved outcomes in 27.8% of patients. The main reasons for the limited success rate were large PVR volumes, difficulties during urination, and persistent urgency/UUI [27]. Patients might not be satisfied with the global response to BoNT-A injection as they expected before treatment. This might explain why only 31% of the patients with idiopathic OAB received more than two BoNT-A injections during long-term follow-up [10].

The safety profile of BoNT-A injection is another concern in patients with idiopathic OAB and urodynamic DO, especially if they are men, have age-associated frailty, have elevated baseline PVR volume, or have combined DO and DU [27]. Patients may develop AUR, difficulties during urination, large PVR volume requiring clean intermittent catheterization (CIC), and subsequent urinary tract infections following BoNT-A injection [28]. Although the adverse effects of BoNT-A resolve with time, patients with idiopathic OAB who can urinate completely at baseline and who later develop adverse events might regard their quality of life to be impaired and the treatment to have failed [27]. A study examined the effects of injection dose, injection site, and injection depth on the occurrence of adverse events following BoNT-A injection. The results indicated that suburothelial injection was as effective as detrusor injection, but trigonal injection had a shorter window of therapeutic efficacy [29]. The occurrence of adverse events did not differ across the different injection subgroups.

This study also reveals that a higher percentage of women had successful treatment outcomes compared with men (59.9% women versus 51.1% men). A previous study reported that men had a higher rate of developing AUR, large PVR volume, and difficulties during urination following BoNT-A injection, mainly because bladder outlet resistance is higher in men without prostatectomy [9]. Because detrusor contractility will be reduced after BoNT-A injection, men with higher urethral resistance usually cannot effectively use abdominal pressure to empty their bladder. Therefore, BoNT-A injection usually has a higher success rate in women, while the rates of subsequent adverse events are higher in men [30]. In this study, the incidence of failed treatment outcomes was similar between the two genders (15.4% in men and 17.4% in women). The findings of this study are in line with those of a previous study that demonstrated that women had a higher rate of successful outcomes and men had a higher rate of AUR after BoNT-A injection for treating OAB [31].

Occult bladder outlet obstruction should be detected cautiously in patients with OAB who are refractory to conventional OAB medications, even when they do not complain of voiding symptoms [32]. The results of this study revealed that patients with urodynamic DO and DU cannot have a successful treatment outcome after BoNT-A injection, and the presence of bladder neck dysfunction, bladder prostatic obstruction, and dysfunctional voiding are likely to decrease the rate of successful outcomes in men and women. Patients with such conditions usually have lower Qmax, larger PVR volumes, and lower VE at baseline; therefore, patients with refractory OAB should be evaluated for the presence of bladder outlet obstruction or DU before BoNT-A injection to avoid large PVR volumes or urine retention after BoNT-A injection, which eventually lead to failed outcomes. Intravesical BoNT-A injection has been reported to have a lower long-term success rate in frail elderly patients and patients with CNS diseases [13,27]. OAB due to CNS disorders such as stroke or Parkinson’s disease may develop urinary retention without reduction in UUI after detrusor BoNT-A injection [33,34]. Among men, those who experienced failed outcomes following BoNT-A treatment were significantly older than those who experienced successful outcomes, suggesting that a high proportion of patients who did not have a successful outcome might be older and frail.

Low detrusor contractility is often associated with urodynamic DO in elderly patients with medical and neurological diseases [35]. BoNT-A injection further lowers detrusor contractility, and the recovery time to the baseline level is higher in these patients compared with patients without DU. Patients with DO and DU usually have lower Qmax and larger PVR volumes, regardless of whether they perceive difficulties during urination [36]. A previous study showed that PVR volume > 200 mL is associated with recurrent urinary tract infection, and CIC is recommended [37]. In patients with DO and Parkinson’s disease, the rate of successful outcomes was only 20%, and the rate of requiring CIC after BoNT-A injection was 28% [15]. The potential adverse events of large PVR and AUR can be prevented in patients with DO and DU by administering a lower dose of BoNT-A (75 U) [38]. In this study, most patients who experienced failed outcomes had a baseline mean PVR volume > 100 mL, further implicating that such patients should be informed about the possibility of developing large PVR volume or AUR after BoNT-A injection. Moreover, they should be recommended to undergo CIC.

The results of logistic regression revealed that low Qmax, small voided volume, and large PVR volume were risk factors for failed treatment outcomes. These results are compatible with a previous study that demonstrated that low baseline VE (<89%) could predict increased PVR volume after intravesical BoNT-A injection [39]. The results of this study suggest that patients with lower baseline VE and lower baseline BCI are likely to have a failed treatment outcome. One previous study had shown that BCI was significantly lower in patients who required CIC after BoNT-A treatment than in those who did not. In women, and a BCI of less than 120 might be predictive of a need for CIC [40]. Therefore, if we regard VE as a proxy for detrusor contractility and urethral resistance, we can use voided volume and total bladder capacity, determined through uroflowmetry or a voiding diary, to calculate VE and to assess the potential risk for failure of intravesical BoNT-A injection. Only patients with a baseline VE > 0.90 can be expected to have a successful treatment outcome at 3 months post-BoNT-A injection.

This study has a few limitations. First, as our study is essentially exploratory, its primary aim was to identify potential predictors of treatment response in DO patients receiving BoNT-A injections. We did not strictly establish a priori hypotheses at the design stage. Although the retrospective design precluded a formal sample size calculation, the large cohort (n = 571) provided adequate power to detect clinically meaningful associations, supporting that our analyses are not purely exploratory. Second, given the retrospective design of this study, a patient’s true perception of the outcome of BoNT-A injections was not recorded from their subjective response to treatment but was rather retrieved from chart records. Third, the patients’ baseline parameters were retrieved from the VUDS reports. These parameters may not reflect the true voiding condition in daily life because the examination was performed with indwelling catheters, not in a natural condition. Fourth, this study is based on single-center-data, which could affect the interpretation and generalizability. The predictors identified in our analyses should therefore be interpreted as preliminary and will require confirmation in larger, prospective cohorts. Nevertheless, this is a large series of data on the treatment outcomes of patients with refractory OAB and urodynamic DO who received intravesical BoNT-A injection. The identified risk factors carry valuable predictive value for patients with refractory OAB who desire to receive BoNT-A injection, such as in patients with urinary incontinence of different lower urinary tract conditions and medical comorbidities such as diabetes mellitus or chronic kidney diseases, post-prostatectomy OAB, and geriatric urinary incontinence. The results of this study provide potential directions for future hypothesis-testing research.

## 5. Conclusions

The results of this study revealed that the following factors could predict successful treatment outcomes in patients with refractory OAB and urodynamic DO: lower baseline Pdet, higher Qmax, larger voided volume, and smaller PVR volume in men; and larger voided volume and smaller PVR volume in women. Patients are likely to have failed treatment outcomes if they have DO and DU (as revealed by a baseline urodynamic study) or bladder outlet obstruction.

## Figures and Tables

**Table 1:** Treatment outcome in male patients with DO after detrusor BoNT-A injection.

Baseline Variables	Successful (*n* = 139)	Improved (*n* = 91)	Failed (*n* = 42)	Total (*n* = 272)	*p* Value
Age	70.9 ± 11.7 ^a^	74.1 ± 10.6	77.4 ± 8.1 ^b^	72.9 ± 11.1	0.002
CVA	9 (6.5%)	7 (7.7%)	3 (7.1%)	19 (7.0%)	0.005
PD	1 (0.7%)	8 (8.8%)	3 (7.1%)	12 (4.4%)	
Dementia	2 (1.4%)	2 (2.2%)	4 (9.5%)	8 (2.9%)	
BPO	0	10 (11%)	2 (4.8%)	12 (4.4%)	0.000
DU/DA	0	23 (25.3%)	23 (54.8%)	46 (16.9%)	0.000
BND	1 (0.7%)	5 (5.5%)	1 (2.4%)	7 (2.6%)	0.062
DV	2(1.4%)	11 (12.1%)	3 (7.1%)	16 (5.9%)	0.003
IDO	127 (91.4%)	74 (81.3%)	34 (81%)	235 (86.4%)	0.050
NDO	11(7.9%)	17 (18.7%)	7 (16.7%)	35 (12.9%)	0.042
BOO	1 (0.7%)	2 (2.2%)	0	3 (1.1%)	0.737
Pdet (cmH_2_O)	30.2 ± 12.3 ^a^ (2–63)	37.1 ± 20.9 ^b^ (4–100)	33.3 ± 28.3 (0–117)	33.0 ± 18.8	0.024
Compliance	50.5 ± 56.3 (3.90–410.0)	61.6 ± 76.4 (0.44–460.0)	39.7 ± 40.9 (0.92–211.0)	52.6 ± 62.2	0.145
Qmax (mL/s)	12.2 ± 5.02 ^a^ (2–29)	8.5 ± 4.3 ^b^ (2–23)	5.91 ± 3.9 ^c^ (0–15)	10.0 ± 5.2	0.000
Vol (mL)	225 ± 103 ^a^ (72–671)	166 ± 104 ^b^ (10–502)	102 ± 84.4 ^c^ (0–382)	186 ± 110	0.000
PVR (mL)	10.3 ± 18.7 ^a^ (0–100)	45.3 ± 66.4 ^b^ (0–480)	110 ± 180 ^c^ (0–900)	37.5 ± 88.0	0.000
CBC (mL)	235 ± 107 (72–671)	212 ± 124 (20–634)	213 ± 189 (28–993)	224 ± 128	0.322
VE	0.96 ± 0.07 ^a^ (0.64–1.00)	0.81 ± 0.21^b^ (0.71–1.00)	0.65 ± 0.33 ^c^ (0.00–1.00)	0.86 ± 0.21	0.000
BOOI	5.76 ± 15.1 ^a^ (−30–47.0)	20.2 ± 22.4 ^b^ (−19–96.0)	21.5 ± 29.3 ^b^ (−10–117)	13 ± 21.6	0.000
BCI	91.5 ± 29.0 ^a^ (34–184)	79.6 ± 30.2 ^b^ (14–178)	62.8 ± 34.5 ^c^ (13–150)	83.1 ± 31.9	0.000

CVA: cerebrovascular accident, PD: Parkinson’s disease, BPO: benign prostatic obstruction, DU: detrusor underactivity, DA: detrusor acontractile, BND: bladder neck dysfunction, DV: dysfunctional voiding, IDO: idiopathic detrusor overactivity, NDO: neurogenic detrusor overactivity, BOO: bladder outlet obstruction, Pdet: detrusor pressure, Qmax: maximum flow rate, Vol: voided volume, PVR: post-void residual, CBC: cystometric bladder capacity, VE: voiding efficiency, BOOI: bladder outlet index, BCI: bladder contractility index. Different superscript letters indicate significant differences (*p* < 0.05) among subgroups based on the Scheffé post hoc test.

**Table 2:** Treatment outcome in female patients with DO after detrusor BoNT-A injection.

Baseline Variables	Successful (n = 179)	Improved (n = 68)	Failed (n = 52)	Total (n = 299)	*p* Value
Age	60.1 ±13.9	62.2 ± 17.7	64.1 ± 15.8	61.3 ± 15.2	0.204
CVA	1 (0.6%)	2 (2.9%)	5 (9.6%)	8 (2.7%)	0.000
PD	1 (0.6%)	0	0	1 (0.3%)	
Dementia	0	3 (4.4%)	4 (7.7%)	7 (2.3%)	
DU/DA	0	22 (32.4%)	19 (36.5%)	41 (13.7%)	0.000
DV	16 (8.9%)	20 (29.4%)	7 (13.5%)	43 (14.4%)	0.000
IDO	177 (98.9%)	63 (92.6%)	43 (82.7%)	283 (94.6%)	0.000
NDO	2 (1.1%)	6 (8.8%)	9 (17.3%)	17 (5.7%)	0.000
Pdet (cmH_2_O)	20.6 ± 10.5 ^a^ (1–54)	27.2 ± 20.1 ^b^ (7.24–358.0)	19.5 ± 14.3 ^a^ (3.81–300.0)	21.9 ± 14.1	0.002
Compliance	68.6 ± 60.4 ^a^ (7.25–427.0)	62.9 ± 68.0 (7.24–358.0)	41.5 ± 49.4 ^b^ (3.81–300.0)	62.6 ± 61.1	0.018
Qmax (mL/s)	17.0 ± 8.4 ^a^ (2.0–55.0)	11.9 ± 6.4 ^b^ (3.0–40.0)	9.3 ± 8.02 ^b^ (0–46.0)	14.5 ± 8.5	0.000
Vol (mL)	255 ± 128 ^a^ (8.0–594)	185 ± 94.7 ^b^ (50–569)	118 ± 104 ^c^ (0–641)	215 ± 128	0.000
PVR (mL)	7.1 ± 17.5 ^a^ (0–162)	76.2 ± 75.9 ^b^ (0–316)	139 ± 175 ^c^ (0–700)	45.8 ± 96.9	0.000
CBC (mL)	262 ± 129 (18–672)	262 ± 116 (50–582)	256 ± 205 (0–834)	261 ± 142	0.968
VE	0.96 ± 0.1 ^a^ (0.24–1.00)	0.74 ± 0.22 ^b^ (0.21–1.00)	0.6 ± 0.32 ^c^ (0.00–1.00)	0.85 ± 0.24	0.000
BOOI	−13.5 ± 20.8 ^a^ (−90–36.0)	3.5 ± 21.2 ^b^ (−28–80.0)	0.96 ± 21.9 ^b^ (−67–62.0)	−7.11 ± 22.4	0.000
BCI	106 ± 42.1 ^a^ (26–295)	86.6 ± 41.3 ^b^ (22–254)	66.0 ± 42.0 ^c^ (5–255)	94.5 ± 44.5	0.000

CVA: cerebrovascular accident, PD: Parkinson’s disease, DU: detrusor underactivity, DA: detrusor acontractile, DV: dysfunctional voiding, IDO: idiopathic detrusor overactivity, NDO: neurogenic detrusor overactivity, Pdet: detrusor pressure, Qmax: maximum flow rate, Vol: voided volume, PVR: post-void residual, CBC: cystometric bladder capacity, VE: voiding efficiency, BOOI: bladder outlet index, BCI: bladder contractility index. Different superscript letters indicate significant differences (*p* < 0.05) among subgroups based on the Scheffé post hoc test.

**Table 3:** Predictive factors of a successful treatment outcome in patients with detrusor overactivity after intravesical BoNT-A injection.

Univariate				Multivariate		
Female	Odd ratio	95% CI	*p*	Odd ratio	95% CI	*p*
Pdet (cmH_2_O)	0.984	0.968–1.000	0.053			
Compliance	1.005	1.000–1.009	0.044	1.008	0.999–1.016	0.081
Qmax (mL/s)	1.134	1.087–1.182	0.000	1.052	0.995–1.112	0.075
Vol (mL)	1.008	1.005–1.011	0.000	1.008	1.004–1.013	0.000
PVR (mL)	0.960	0.949–0.971	0.000	0.957	0.945–0.969	0.000
CBC	1.000	0.999–1.002	0.871			
VE	2.549	1.911–3.265	0.000			
BOOI	0.962	0.949–0.975	0.000			
BCI	1.018	1.011–1.026	0.000			
Male	Odd ratio	95% CI	*p*	Odd ratio	95% CI	*p*
Pdet (cmH_2_O)	0.983	0.970–0.997	0.014	0.966	0.947–0.985	0.001
Qmax (mL/s)	1.259	1.174–1.350	0.000	1.147	1.053–1.248	0.002
Vol (mL)	1.008	1.005–1.010	0.000	1.006	1.002–1.009	0.002
PVR (mL)	0.973	0.963–0.982	0.000	0.971	0.960–0.982	0.000
CBC	1.001	1.000–1.003	0.135			
VE	2.290	1.770–2.961	0.000			
BOOI	0.960	0.946–0.975	0.000			
BCI	1.019	1.010–1.028	0.000			

Pdet: detrusor pressure, Qmax: maximum flow rate, Vol: voided volume, PVR: post-void residual, CBC: cystometric bladder capacity, VE: voiding efficiency, BOOI: bladder outlet index, BCI: bladder contractility index.

**Table 4:** Receiver operation curves for area under curves (AUC) and cut-off values to predict patients with overactive bladder who might have an unsatisfactory (improved + failed) treatment outcome.

Female	AUC	Cut-Off Value	Sensitivity	Specificity
Qmax (mL/s)	0.749	12.5	69.2%	70.4%
Compliance	0.624	37.17	69.3%	55.8%
Vol (mL)	0.742	229.5	85%	51.4%
PVR (mL)	0.816	35	61.7%	94.4%
VE	0.825	0.895	72.3%	89.9%
Male	AUC	Cut-Off value	Sensitivity	Specificity
Qmax (mL/s)	0.772	8.5	62.4%	79.9%
Vol (mL)	0.733	149	62.4%	75.5%
PVR (mL)	0.702	35	45.1%	90.6%
VE	0.726	0.8008	48.1%	97.8%

AUC: area under curve, Qmax: maximum flow rate, Vol: voided volume, PVR: post-void residual, VE: voiding efficiency.

## Data Availability

Data are contained within the article.
